# Baicalin: a potential therapeutic agent for acute kidney injury and renal fibrosis

**DOI:** 10.3389/fphar.2025.1511083

**Published:** 2025-01-22

**Authors:** Xiaoming Li, Rui Xu, Dan Zhang, Ji Cai, He Zhou, Tao Song, Xianyao Wang, Qinghong Kong, Liujin Li, Zhaohui Liu, Zhixu He, Zhengzhen Tang, Jun Tan, Jidong Zhang

**Affiliations:** ^1^ Department of Immunology, Zunyi Medical University, Zunyi, China; ^2^ Special Key Laboratory of Gene Detection and Therapy of Guizhou Province, Zunyi Medical University, Zunyi, China; ^3^ Zunyi Medical University Library Administrative Office, Zunyi, China; ^4^ Guizhou Provincial College-Based Key Lab for Tumor Prevention and Treatment with Distinctive Medicines, Zunyi Medical University, Zunyi, China; ^5^ Department of Otolaryngology, Affiliated Hospital of Zunyi Medical University, Zunyi, China; ^6^ Collaborative Innovation Center of Tissue Damage Repair and Regeneration Medicine, Zunyi Medical University, Zunyi, China; ^7^ Department of Pediatrics, The First People’s Hospital of Zunyi, Third Affiliated Hospital of Zunyi Medical University, Zunyi, China; ^8^ Department of Histology and Embryology, Zunyi Medical University, Zunyi, China

**Keywords:** acute kidney injury, renal fibrosis, herbal medicine, baicalin, mechanism

## Abstract

Acute kidney injury (AKI) is a common critical clinical disease that is linked to significant morbidity, recurrence, and mortality. It is characterized by a fast and prolonged loss in renal function arising from numerous etiologies and pathogenic pathways. Renal fibrosis, defined as the excessive accumulation of collagen and proliferation of fibroblasts within renal tissues, contributes to the structural damage and functional decline of the kidneys, playing a pivotal role in the advancement of Chronic Kidney Disease (CKD). Until now, while continuous renal replacement therapy (CRRT) has been utilized in the management of severe AKI, there remains a dearth of effective targeted therapies for AKI stemming from diverse etiologies. Similarly, the identification of specific biomarkers and pharmacological targets for the treatment of renal fibrosis remains a challenge. Baicalin, a naturally occurring compound classified within the flavonoid group and commonly found in the Chinese herb Scutellaria baicalensis, has shown a range of pharmacological characteristics, such as antioxidant, anti-inflammatory, antifibrotic, antitumor and antiviral effects, as evidenced by research studies. Research shows that Baicalin has potential in treating kidney diseases like AKI and renal fibrosis. This review aims to summarize Baicalin’s progress in these areas, including its molecular mechanism, application in treatment, and absorption, distribution, metabolism, and excretion. Baicalin’s therapeutic effects are achieved through various pathways, including antioxidant, anti-inflammatory, antifibrosis, and regulation of apoptosis and cell proliferation. Besides, we also hope this review may give some enlightenment for treating AKI and renal fibrosis in clinical practice.

## Graphical Abstract

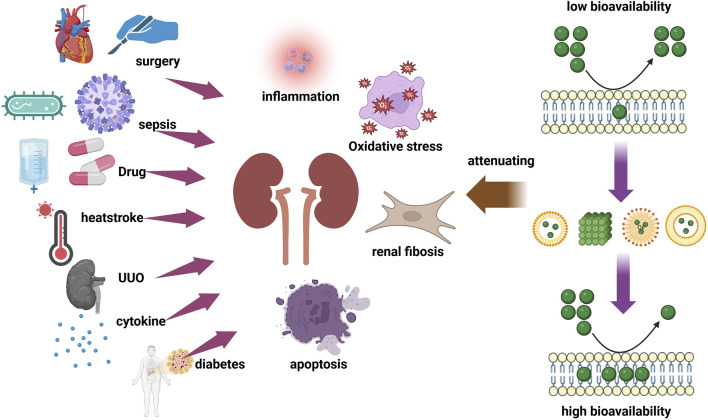

## 1 Introduction

Acute kidney injury (AKI) is defined by a sudden drop in renal function, including an increase in serum creatinine (SCr) (≥0.3 mg/dL within 48 h, or ≥1.5 times baseline), or a urine volume of <0.5 mL/kg/h for 6 h (Turgut et al., 2023). 30%–60% of critically sick patients worldwide suffer from AKI, which is linked to acute morbidity and mortality (Hoste et al., 2018). AKI is a clinical syndrome caused by multiple etiologic factors and can result from various kinds of injuries, such as exposure to nephrotoxins, decreased renal perfusion, urinary obstruction, or nephrogenic renal disease, causing a dramatic decrease in renal function in the short term, manifested as a decrease in the glomerular filtration rate (GFR) with retention of nitrogen products, such as SCr and blood urea nitrogen (BUN), and disturbances in the water, electrolyte, and acid-base balances, which can be severe and can lead to multiple Systemic complications can occur in severe cases (Ronco et al., 2019; Erfurt et al., 2023). Failure to recognize, intervened and treated early, it can cause serious damage to the kidneys, progress to chronic kidney disease (CKD), and increase the risk of cardiovascular events, and even lead to death (See et al., 2019; Kurzhagen et al., 2020). However, the current treatment of AKI is only supportive, with limited therapeutic options, and short-term mortality remains high.

CKD is usually caused by an assortment of factors, including hypertension, diabetes, and glomerulonephritis (Rapa et al., 2019). These pathologies lead to glomerular and tubular damage, which triggers an inflammatory response and cellular damage. Over time, the degree of kidney damage increases and the fibrotic process begins to develop as collagen begins to be deposited, gradually replacing the healthy kidney tissue, and this will eventually lead to further decline in renal function and deterioration of CKD (Hung et al., 2021; Huang et al., 2023).

Currently, the main means of clinical treatment for AKI is to correct reversible etiological and pre-renal factors as soon as possible at the onset of AKI, including volume expansion, maintenance of hemodynamic stability, discontinuation of drugs affecting renal perfusion and so on, whereas glucocorticoids and/or immunosuppressant therapy are commonly used for AKI secondary to glomerulonephritis. Drug usage is not risk-free, though. Oral glucocorticoids have been shown in a cohort study to substantially increase the risk of adrenal insufficiency, Cushing syndrome, and death (Mebrahtu et al., 2019).

Endothelin receptor antagonists (ERAs), sodium-glucose transporter-2 (SGLT-2) inhibitors, dipeptidase transferase-4 (DPP-4) inhibitors, salt-corticosteroid receptor antagonists, and N-acetyl-sericyl-aspartate-lysine (Ac-SDKP) are among the numerous pharmaceutical agents presently undergoing clinical trials for the treatment of diabetic nephropathy. These substances have been shown to offer defense against increased renal inflammatory and fibrotic responses in addition to preclinical decrease in renal function (Bhatia and Srivastava, 2023). Traditional treatments for diabetic nephropathy (DN) include renin angiotensin aldosterone system (RAAS) inhibitors such as angiotensin-converting enzyme inhibitors (ACEIs) and angiotensin II receptor blockers (ARBs), which are first-line agents that are effective in reducing the incidence of end-stage renal disease (Srivastava et al., 2020a). In DN animal models, ACEIs and ARBs fulfill distinct roles. Specifically, ACEIs mitigate renal fibrosis by attenuating dipeptidyl peptidase-4 (DPP-4) and transforming growth factor-beta (TGFβ) signaling pathways, a function not observed with ARBs (Srivastava et al., 2020b). Heart failure, coronary artery disease, essential hypertension, and CKD are among the clinical conditions for which ACEI and ARBs can be utilized as antihypertensive medications. However, ACEI and ARBs have slightly different mechanisms of action. By decreasing the generation of angiotensin II, vasoconstriction, and aldosterone secretion, ACEI lower blood pressure and cardiac burden. ARBs, on the other hand, directly prevent angiotensin II from binding to its receptors, which may occasionally lead to less adverse effects, such as coughing (Matchar et al., 2008), angioedema (Makani et al., 2012). Therefore, the search for an effective therapeutic drug is imperative.

One of the most widely used multipurpose herbs or medicinal plants for treating bacterial and viral infections, allergies, and inflammation is Scutellaria baicalensis (Yoon et al., 2009; Mir-Palomo et al., 2016; Jung et al., 2012). Baicalin (C21H18O11), a flavonoid extracted from Scutellaria baicalensis (SR) dried roots, has been evaluated in multiple types of AKI. The pharmaceutical industry has become increasingly interested in baicalin due to its superior biological effect. Baicalin has also been reported to exhibit various biological properties, including anti-inflammatory (Fu et al., 2021), antioxidant (Ganguly et al., 2022), antiviral (Feng H. et al., 2022) and anti-tumor (Song et al., 2022). Other pharmacological effects of this compound include antibacterial activity, purgative and detoxification (Bai et al., 2018). Experimental studies have demonstrated that baicalin is protective against renal damage caused by various stimulation, including renal ischemia, nephrotoxic drugs and sepsis while being able to effectively intervene in the occurrence of renal fibrosis (Wang et al., 2015; Le et al., 2021; Shi et al., 2019; Zhang S. et al., 2020). Therefore, this article mainly reviews its renoprotective effects of baicalin. We believe that baicalin may be a promising drug to the treat patients with AKI and renal fibrosis. Currently, most clinical therapeutic drugs cause further kidney damage to varying degrees. In order to prevent AKI from occurring and to create successful preventive and therapeutic methods, it is imperative that safe and effective natural active components be found, given the current clinical status of AKI treatment.

## 2 Baicalin

Baicalin (C21H18O11) is a natural herbal compound of flavonoids. It is utilized extensively in traditional Chinese medicine and is mostly found in Scutellaria baicalensis, a traditional Chinese medication. 6 polysaccharides and 126 small molecule compounds have been identified from SR thus far (Wang Z. L. et al., 2018). Among them, baicalin, wogonoside, baicalein and wogonin are recognized as the most effective drug candidates. The chemical symbol for baicalin is 7-glucuronic acid, 5,6-dihydroxyflavone, and its molecular weight is 446.4 g/mol. The structure of baicalin has a number of peculiarities, containing a glycosidic group (glycoside) and a flavonoid backbone. First, the glycosidic portion of baicalin is formed by linking glucose and baicalein. This linkage is known as the glycosidic bond, which makes baicalin highly stable and easy to store. For example, one study showed that eight flavonol O-glycosides exhibited antioxidant activity (Kimura H. et al., 2017). In addition, the flavonoid backbone of baicalin is composed of two benzene rings connected by three carbons. This special backbone structure confers many pharmacological and biological activities to baicalin. The phenolic hydroxyl group and carbonyl group of baicalin play important roles in pharmacological activities. For instance, a study showed that the phenolic hydroxyl group of flavonoids, including baicalin, can have a scavenging effect on hydroxyl radicals (Chen et al., 2002). Due to its unique chemical structure, baicalin has many pharmacological activities. It is considered to have anti-inflammatory, antioxidant, antibacterial, antitumor, and antiallergic effects, making baicalin a natural pharmaceutical compound that has been widely studied and applied.

### 2.1 Absorption

Baicalin itself cannot be directly absorbed by the intestinal tract and is first hydrolyzed by intestinal bacteria to its glycoside baicalein (Akao et al., 2000). Using the *in situ* absorption approach, a preliminary assessment of the absorption sites of baicalin in rats’ stomachs and various intestine segments revealed two distinct sites of absorption in one research. Baicalin was absorbed in two different ways: first, it was absorbed in the upper intestine, probably directly; and second, it was absorbed as glycosides in the colon (Lu et al., 2007). One of the most significant techniques for researching medication absorption in rats is *in situ* perfusion. In a rat study, the authors performed *in situ* perfusion in rats with and without ligated bile ducts, and the experiments’ findings from 2006 demonstrated that baicalin absorbed badly in the small intestine and colon and somewhat in the stomach, suggesting that baicalin was unable to effectively pass through the intestinal epithelium (Taiming and Xuehua, 2006).

### 2.2 Distribution

Baicalin can accumulate in various tissues. The largest quantity of baicalin was detected in the kidneys, and the drug’s subsequent concentration in each of the main organs in the following order: kidneys > lungs > liver > spleen, according to a tissue distribution study conducted after injection. Following an intravenous injection of liposomal baicalin, the lungs exhibited the highest concentration of the medication. Subsequently, the concentration of the drug declined in the following sequence in the major organs and tissues: kidney > spleen > liver > lungs (Wei et al., 2016). Another finding was that baicalin in the preparation of Huanglian Xieyu Tang was rapidly distributed in the lungs and accumulated at high levels in the lungs (Zhu et al., 2013). In the kidney, the distribution of scutellarin and its metabolite scutellarein isolated from Scutellaria baicalensis showed that the two were mainly distributed in the renal cortex and medullary regions with high abundance, suggesting that scutellarin may have the potential to prevent and treat kidney diseases (Wang T. et al., 2021).

### 2.3 Metabolism

With regard to gastrointestinal hydrolysis, enterohepatic circulation, carrier-mediated transport, and complex metabolism, baicalin possesses a distinct pharmacokinetic profile. Once baicalin is absorbed, the interaction between baicalin and its aglycone baicalein will occur in the body. Studies in rats have demonstrated that baicalin is rapidly converted to baicalein in the gut through the enzymatic activity of β-glucuronidase, a process contingent upon the presence of gut microbiota. Then, once baicalin enters the systemic circulation, it is converted back to baicalein by Uridine diphosphate glucuronosyltransferase (UGT) (Akao et al., 2000; Noh et al., 2016). 18.7% of the baicalin that was administered orally entered the enterohepatic circulation. These findings demonstrate that a substantial amount of baicalin is subjected to first-pass glucuronidation and that the enterohepatic circulation significantly influences the amount of baicalin that is exposed to rats (Xing et al., 2005a). Wistar rats’ plasma contained baicalein 6-O-β-dlucopyranuronoside (B6G) as the major metabolite after oral baicalin treatment at 20 mg/kg of dosage, and its level was greater than that of baicalin (Akao et al., 2013). Furthermore, the existence of baicalin isomers in plasma following oral treatment suggests that baicalin is at least partially converted to baicalein prior to absorption and then to its conjugated metabolite in rats (Xing et al., 2005b). In recent years, in order to obtain more metabolites, the metabolites of baicalin were studied by linear ion trap-orbitrap mass spectrometry, and 32 metabolites in all were found and described. Also, The corresponding reactions of baicalin *in vivo* were also found, such as methylation, methoxylation, hydrolysis, hydroxylation, glucuronic acid binding, sulfate binding and complex reactions (Zhang et al., 2015), indicating that baicalin has a complicated metabolism and that these isomers could greatly enhance baicalin’s benefits if they have biological activity.

### 2.4 Excretion

In the body, baicalin is first absorbed as baicalein and subsequently converted to baicalin. It is possible for the colon to excrete some of the baicalein that is generated from absorbed or intravenously administered baicalein (Taiming and Xuehua, 2006). Meanwhile, in a previous study, using the *in situ* jejunal loop technique and *in vitro* jejunal ectopic capsule assay, It was shown that multidrug resistance binding protein 2 (MRP2) significantly processed baicalein in intestinal mucosal cells to produce baicalin, which was then eliminated into the intestinal lumen (Akao et al., 2004). It is important to note that rats and humans dispose of baicalin quite differently. Notably, the UGT activity of rat jejunum microsomes against baicalin is comparable to that of liver microsomes, even though the liver is traditionally regarded as the primary site for xenobiotic metabolism (Akao et al., 2004). Furthermore, an *in vitro* absorption experiment utilizing everted rat jejunal sacs revealed that baicalin emerged outside the sac rather than inside it, whereas very little of the baicalein absorbed was transported to the inner (serosal) side of the sac. Eisai hyperbilirubinemia rats (EHBR) with hereditary MRP2 deficiency exhibited a considerably decreased jejunal sac effection rate (56.4%) in comparison to wild-type Sprague-Dawley rats, indicating that baicalin efflux may be mediated by MRP2. The human colonic adenocarcinoma cell line Caco-2 is used to study the absorption and disposition of drugs in the human gut, although it is limited in the expression of certain transporters and metabolic enzymes. Baicalein can be transported bidirectionally (from the apex to the basal side and from the basal side to the apex) in a single-layer model of Caco-2 cells, while the levels of the glucuronides formed are very low (Ng et al., 2004). In addition, baicalin was mainly accumulated on the basal side, suggesting that MRP3 may be involved in baicalin transport (Zhang et al., 2005). Furthermore, research has demonstrated that the primary transport medium for the body’s absorption and utilization of flavonoids (including baicalin, catechin, quercetin, etc.) is human serum albumin (HSA). The degree to which baicalin binds to human serum albumin determines its transit rate and volume distribution inside the host. Specifically, the relative absorption rate of baicalin is approximately 65%, as indicated by the area under the time-concentration curve (AUC_ ∞ _) (Wen et al., 2023). This difference may be related to differences in intestinal flora composition, metabolic enzyme activity, and transporter expression between humans and rats, which together affect drug absorption and metabolism processes. It has also been reported that enzymes in the digestive tract (e.g., β-glucosidase or lactase chlorin hydrolase (LPH) are involved in the hydrolysis of flavonoids, which suggests to us that this enzyme may be involved in the hydrolysis of baicalin (Murota et al., 2018). A study that included ten healthy male volunteers also discovered that baicalin was recovered from urine at doses of 5.0, 10.0, and 20.0 mg/mL, with baicalin recoveries from urine of 105.9%, 101.1%, and 108.4%, respectively, after oral administration of the drug. Correspondingly. Baicalin was recovered in 105.9%, 101.1%, and 108.4% of the urine samples, suggesting that urine is another route by which baicalin can be eliminated (Lai et al., 2003).

## 3 Protective effect of baicalin on AKI

AKI has multiple causes, which fall into three primary categories: pre-renal, renal, and post-renal, according to the anatomical site where the cause occurs. In terms of causes, the causes include drugs (Privratsky et al., 2018), sepsis (Huang J. et al., 2019), obstruction, and Renal hypoperfusion, etc. Regardless of the cause of AKI, the specific pathomechanisms include inflammatory response, oxidative stress, immune dysregulation, apoptosis, and mitochondrial dysfunction, among others (Kumar, 2018; Sureshbabu et al., 2015; Kimura T. et al., 2017; Thomas et al., 2022). The main manifestation is ischemic damage to the kidney’s proximal tubules, with detachment of tubular epithelial cells and brush border and eventual loss of proximal tubular function (Makris and Spanou, 2016). The absence of a “gold standard” for calculating AKI and the use of various benchmarks to define the condition contribute to the varying incidence of AKI (Chang-Panesso, 2021). An abrupt (within hours to days) drop in glomerular filtration rate that causes the retention of nitrogenous waste products (such as BUN and SCr) in the blood plasma is referred to AKI, which replaces early acute renal failure. It occurs in 5.0%–7.5% of hospitalised patients and 50%–60% of critically ill patients (Rodrigues et al., 2013; Gameiro et al., 2020; Hoste et al., 2015). Throughout the past few years, a number of experimental models of AKI have been used to examine the nephroprotective benefits of baicalin (Le et al., 2021; Shi et al., 2019).

**FIGURE 1:**
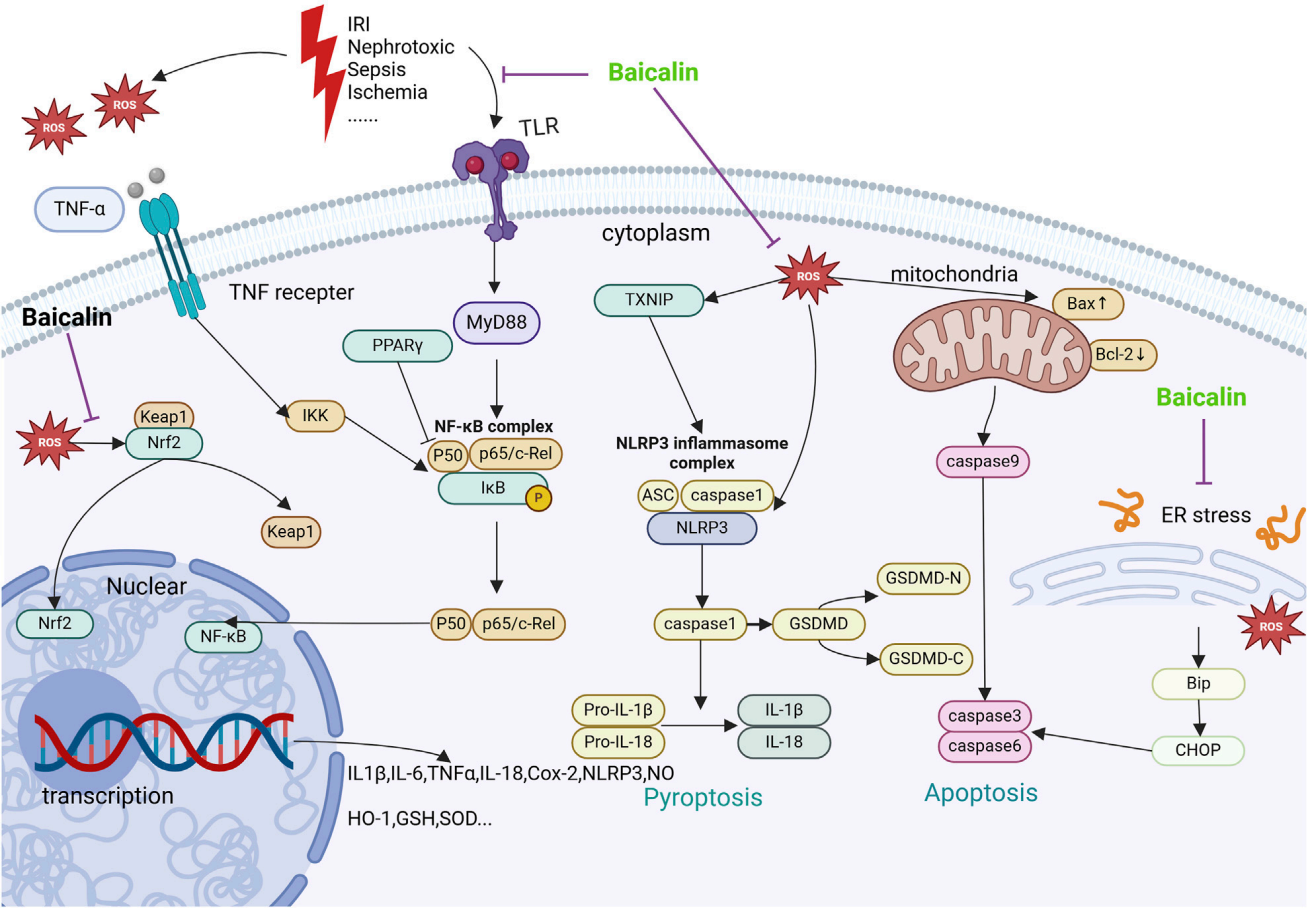
Mechanisms by which baicalin intervenes in AKI. This figure summarizes the molecular pathways by which baicalin treats AKI. Receptors such as TNFR, TLR are subjected to nephrotoxic stimuli such as lipopolysaccharide I/R and inflammatory factors. Furthermore, mitochondrial quality control mechanisms and membrane potential are directly impacted by hypoxia and ischemia-reperfusion, which results in the generation of ROS. The aforementioned receptors’ activation along with the creation of intracellular ROS might set off subsequent cascades that worsen inflammation, apoptosis, and renal damage. Baicalin targets Nrf2 and activates the expression of anti-aging chemokines to enhance cellular tolerance to oxidative stress. By blocking the NF-κB pathway via TLR2/4 and TNFR, baicalin inhibits the inflammatory response and prevents the synthesis of pro-inflammatory proteins. Baicalin also decreases apoptosis by preventing endoplasmic reticulum stress, which is known to alleviate AKI through ROS/NLRP3/Caspase-1/GSDMD Pathway-Mediated proptosis.

As shown in Table 1, the studies *in vivo* and *in vitro* of the herbal medicine baicalin for the treatment of AKI are listed. The studies include AKI caused by nephrotoxic drugs and toxins, ischemia/reperfusion contrast agents, sepsis, etc. Mechanistically, Baicalin may lessen oxidative stress, damage to mitochondria and other organelles, inflammation, and apoptosis, among other cytoprotective mechanisms, to lessen AKI (Figure 1). A more thorough explanation of these mechanisms can be found below.

**TABLE 1:** The studies of baicalin intervention AKI *in vivo* and *in vitro*.

Type	Animal/Cell	Experimental model	Dosages	Pharmacological effect	Mechanism (marker)	References
AKI Induced by nephrotoxic drugs (drug-induced)	HK-2 cells	H_2_O_2_-induced AKI	Baicalin (100 μmol/L)	Anti-apoptosis, antioxidation	BiP↓, CHOP↓, Nrf2↑, ROS↓, GSH/GSSG↓, Caspase-3↓	Zhang et al. (2020b)
	Mice	Aristolochic acid I-induced AKI	Baicalin (80 and 160 mg/kg)	Reduced BUN and SCr, improved the extensive tubular necrosis and tubular dilatation	BUN↓, SCr↓, CYP1A1↑, CYP1A2↑	Fu et al. (2021)
	Mice	Lead-induced AKI	Baicalin (12.5, 25, 50 mg/kg)	Anti-inflammatory, anti-apoptosis	SOD↑, GSH-Px↑, MDA↓, Bax↓, Bcl-2↑	Kelley et al. (2019)
	HK-2 cells	Contrast-induced AKI	Baicalin (10 mg/mL)	Antioxidant, anti-inflammatory	NLRP3↓, IL-1β↓, IL-18↓, ASC↓, Caspase-1↓, GSDMD↓, LDH↓, SOD↑, MDA↓, ROS↓	Rodrigues et al. (2013)
AKI Induced by Sepsis (infection induction)	ICR mice	LPS-induced AKI	Baicalin (74 mg/kg/d)	Reduced histopathological changes, anti-inflammatory, antioxidant	SCr↓, TNF-α↓, IL-1β↓, IL-6↓, IL-10↓, NO↓, MDA↓, ALT↓, prolylate synthase CS↑, ketoacid kinase PK↑	Gameiro et al. (2020)
	HK-2 cells	LPS-induced AKI	Baicalin (5, 15, 25, 50, 75 μmol/L)	Inhibited inflammation response	TNF-α↓, IL-6↓, IL-1β↓, iNOS↓, COX-2↓, NF-κB65↓, TXNIP↓, NLRP3↓	Lim et al. (2012)
	C57BL/6 mice	cLP-induced AKI	Baicalin (200 mg/kg/d)	Reduced BUN and SCr, anti-apoptosis	BUN↓, SCr↓, Bax↓, Bcl2↑	Li et al. (2020)
	ICR mice	cLP-induced AKI	Baicalin (50 and 100 mg/kg)	Anti-apoptosis, decreased the pathological injury of renal tissue	BUN↓, SCr↓, c-FLIP↑, pNGAL↓,pKIM-1↓	Ganguly et al. (2022)
AKI Induced by Ischemia	Wistar rats	Renal ischemia-reperfusion injury	Baicalin (10 and 100 mg/kg)	Reduced BUN and SCr, improved renal histopathology scores, Anti-apoptosis, antioxidant, anti-inflammatory	BUN↓, SCr↓, MDA↓,SOD↑, TLR2/4↓, MyD88↓, p-NF-κB↓, p-IκB↓, IκB↑, caspase-9↓, caspase-3↓, Bax,↓, Bcl-2↑	Ramesh and Reeves (2002)
	Sprague-Dawley rats	75-min cardiopulmonary bypass (CPB) and 45-min cardiac arrest (CA) to establish an AKI model in rats	Baicalin (100 and 200 mg/kg)	Reduced BUN and SCr, antioxidant, anti-inflammatory	BUN↓, SCr↓, Kim1↓, NGAL↓, MDA↓, MPO↓, SOD↑, GSH↑ IL-18↓, iNOS↓, Nrf2↑, HO-1↑	Feng et al. (2022a)
AKI Induced by other way	Sprague Dawley rats	Preeclampsia (PE) rat disease model	Baicalin (50, 100, 150 mg/kg/d)	Attenuated acute injury symptoms, anti-apoptosis	Bcl-2↑, XIAP↑, Caspase-3↓, Caspase-6↓, Caspase-9↓, AT1↓	Lin et al. (2014b)
	Sprague-Dawley rats	Sodium taurocholate –introduce SAP	Baicalin (100 mg/kg/h)	Reduced BUN and SCr, improved renal, histopathological scoring, anti-inflammatory	BUN↓, SCr↓, NO↓, TNF-α↓, IL-6↓, ET-1↓, Bax↑,Bcl-2↓, PLA_2_↓	Zhao et al. (2021)
	Sprague-Dawley rats	Sodium taurocholate –introduce SAP	Baicalin (100 mg/kg/h)	Improved renal histopathological scoring	AMY↓, NO↓, MDA↓, TNF-α↓	Jang et al. (2017)
	Sprague-Dawley rats	Heatstroke induced AKI model	Baicalin (10, 20, 40 mg/kg)	Reduced BUN and SCr, anti-inflammatory	BUN↓, SCr↓, IL-1β↓, TNF-β↓	Kønig et al. (2019)
	Fischer 344 aged rats	LPS-induced AKI	Baicalin (10 mg/kg/d)	Anti-inflammatory	VCAM-1↓, IL-1β↓, IL-6↓, PPARγ↑, NF-κB↓	Hoste et al. (2015)

### 3.1 Baicalin prevents AKI by reducing the inflammatory response

The physiological process of inflammation serves to shield the body against acutely harmful stimuli like ischemia, poisons, or viruses, and plays a defensive role in injury or infection (Saade et al., 2021). Inflammation is closely related to AKI, an inflammatory state can cause AKI, which in turn can exacerbate the inflammatory response. Indeed, a number of ischemia, toxic, or immune disorders can harm renal cells by causing inflammation and cell death, which subsequently damages other organs and ultimately results in total failure (Feng Q. et al., 2022). Pro-inflammatory cytokines, chemokines, adhesion molecules, different growth factors, and nuclear factors are some of the molecular characteristics of inflammation that are linked to the inflammatory response. Minimizing the detrimental effects of cytokines on the kidneys is crucial since the decline in renal function during AKI typically causes cytokine concentrations to rise in tandem with a decrease in plasma cytokine clearance (Andres-Hernando et al., 2012). AKI trauma patients had much greater levels of IL-1, IL-8, IL-6 and MCP-1 early in life compared to trauma patients without AKI (Ma et al., 2021). Inflammatory mediators such as inflammatory factors (TNF-α, TGF-β, IL-6, IL-1β, IL-18), chemokines (CCL2), and adhesion factors (ICAM-1,MCP-1 and P-selectin) are induced when kidney injury occurs, which facilitates the recruitment of leukocytes to the kidney and the arrival of macrophages, neutrophils, and lymphocytes at the site of injury (McWilliam et al., 2021). According to recent research, TNF-α, IL-6, and IL-1β have been found to be significant participants in the pathogenesis of renal injury (Ramesh and Reeves, 2002). And inhibition of these inflammatory cytokines attenuates renal tissue damage. Therefore, early anti-inflammatory treatment can improve renal function. By inhibiting the manufacture of inflammatory factors and preventing the inflammatory pathway, baicalin can provide protection against AKI. As evidenced by a notable decrease in IL-10 levels and a considerable increase in TNF-α, IL-8, and IL-6 levels in renal tissue, cisplatin was found to induce a strong pro-inflammatory response in rat renal tissue in one investigation (Kumar et al., 2017). And in several studies, Baicalin demonstrated its ability to suppress ROS release, which raised oxidative stress in cells, released more malondialdehyde (MDA) and superoxide dismutase (SOD), and reduced TNF-α, IL-6, IL-8, and IL-1β levels (Li et al., 2022; Liao et al., 2016; Lim et al., 2012). Academic circles largely recognize the nuclear transcription factor κB (NF-κB) pathway as a significant pro-inflammatory signaling pathway, mostly because of NF-κB’s critical function in controlling the expression of pro-inflammatory genes such adhesion molecules, cytokines, and chemokines (Lawrence, 2009). By coordinating inflammatory processes that aid in the onset and progression of AKI, NF-κB plays a essential role in the pathophysiology of the condition (Liu and Dong, 2021). NF-κB binds to IκB protein in an inactivated state under normal conditions. When cells are stimulated internally and externally, activation of proximal signal bridging proteins leads to activation of IκB kinase and degradation of IκB proteins by phosphorylation ubiquitination, and thus NF-κB is released, translocated to the nucleus and binds to particular target genes, initiating transcription of the target genes and further inducing an acute inflammatory response. The activation of classical NF-κB causes the active immune system to create pro-inflammatory cytokines, which in turn trigger an inflammatory response (Oeckinghaus et al., 2011). Conversely, these pro-inflammatory cytokines activate classical NF-κB (Yu et al., 2020). This leads to a vicious circle. Studies have shown that baicalin significantly reduced the number of CD68^+^ macrophages and CD3^+^ lymphocytes, in addition to affecting NF-κB signaling by downregulating IκB expression and upregulating TNF-α, NLRP3 and P65 expression (Zhang X. T. et al., 2020). Baicalin inhibits the TLR4/MyD88/NF-κB signaling cascade in an ischemia-reperfusion injury (IRI) model, hence reducing the synthesis of pro-inflammatory proteins brought on by renal ischemia-reperfusion injury (IRI). Furthermore, it suppresses apoptosis by reducing cleaved caspase-9 levels (Lin et al., 2014a). An essential component of the innate immune system, the NLRP3 inflammasome promotes caspase-1 activation and the release of pro-inflammatory cytokines, such as IL-1β and IL-18, in response to microbial infections and cellular injury (Kelley et al., 2019). In cisplatin-induced AKI (CI-AKI) models that are both *in vivo* and *in vitro*, renal damage and apoptosis have been shown to be aggravated by the activation of the NLRP3 inflammasome and the ensuing generation of IL-1β and IL-18 (Shen et al., 2016; Lin et al., 2019). In contrast, following baicalin therapy, the pro-inflammatory factors IL-1β and IL-18 as well as the amount of active caspase-1 were reduced due to the inhibition of NLRP3 inflammasome activation (Li et al., 2022; Sun et al., 2020). In conclusion, baicalin has a potent anti-inflammatory action that may prevent AKI by lowering immune cell activation and infiltration as well as the synthesis and release of inflammatory mediators.

### 3.2 Baicalin prevents AKI by reducing oxidative stress

An imbalance in the body between oxidation and antioxidants is identified as oxidative stress. The overabundance of ROS is acknowledged as a critical element contributing to the development of oxidative stress. Differential intracellular oxidative stress levels cause intracellular oxidative components to be misregulated, which in turn causes the production of oxidative markers like SOD and MDA, which promotes the process of apoptosis (Hajam et al., 2022). In renal cells, oxidative stress causes a number of harmful outcomes, including DNA damage, lipid peroxidation, protein modification, pro-inflammatory and pro-fibrotic pathway activation, and apoptosis induction (Emma et al., 2016). A range of antioxidant defense mechanisms exist within cells for scavenging free radicals and maintaining oxidation-reduction balance. These include antioxidant enzymes (e.g., SOD, GSH) and non-enzymatic antioxidant substances (e.g., glutathione, vitamin C, vitamin E, etc.). Research has indicated a significant correlation between oxidative stress and AKI. The aetiology of increased oxidative stress and consequent ischemic renal cell injury is linked to the direct nephrotoxic effects of contrast agents and cisplatin on renal tubular epithelial cells as well as the physiological alterations brought on by the release of vasoactive molecules (Chandiramani et al., 2020; Holditch et al., 2019). ROS disrupt a variety of signaling pathways, such as PI3K, MAPK, Nrf2, iron metabolism, and cell death, if they are not properly neutralized and strong antioxidant baicalin controls oxidative stress in cells through both direct and indirect means, including scavenging ROS and raising the activity of antioxidant enzymes, which prevents AKI (Li et al., 2022; Lin et al., 2014b). Large-scale production of ROS in the AKI model causes direct oxidative damage to the lipids and proteins in the mitochondria, which reduces the permeability of the mitochondrial membrane by interfering with electron transfer chain (ETC) function and raising mitochondrial bioenergetics (Zhao et al., 2021). Significantly reduced lipid peroxidation MDA increased superoxide dismutase SOD levels after baicalin treatment (Lin et al., 2014a; Zhang et al., 2017). In contrast-induced AKI, it also showed strong antioxidant effects, including lowering ROS, MDA, and increasing SOD levels (Li et al., 2022). Research has indicated that exposure to lead increases oxidative stress (Lin et al., 2019; Sun et al., 2020) and that SOD, GSH-Px, and MDA are targets of lead (Hsu and Guo, 2002). Exposure to lead affects the levels and activity of antioxidant enzymes, thereby damaging cells and tissues. In addition, mice develop a dose-dependent accumulation of lead, primarily in the kidneys (Yu et al., 2022). In contrast, following baicalin therapy, the baicalin group’s SOD and GSH-Px activity rose, and baicalin dose-dependently reduced the elevated MDA levels. Nrf2, a transcription factor, is typically located in the cytoplasm where it binds to the actin-associated Keap1 protein and undergoes normal degradation. This relationship is broken under oxidative stress circumstances, which leads to the translocation of Nrf2 into the nucleus and the subsequent upregulation of the synthesis of cytoprotective enzymes, including HO-1 and SOD (Li et al., 2020). It is well established that the Nrf2-associated signaling pathway provides protection against acute kidney injury brought on by a variety of circumstances (Wang Z. et al., 2021). In rats with AKI from cardiac surgery, The Nrf2 defense mechanism was significantly activated by baicalin pretreatment, as evidenced by increased kidney levels of Nrf2 and downstream aging inhibitor enzymes (e.g., HO-1) (Shi et al., 2019). This implies that baicalin activates Nrf2 oxidative stress, which impairs the defense mechanism against acute kidney injury caused by cardiac surgery. Thus, baicalin has therapeutic potential to antagonize oxidative stress in AKI of various etiologies.

### 3.3 Baicalin prevents AKI by reducing apoptosis

Given that tubular damage can result in a sharp reduction in renal function, it has been observed that tubular epithelial cells injury in sepsis-associated AKI(SA-AKI) (Zhang et al., 2024). Notably, when renal tubular injury is severe, this damage will be irreversible. Although preventive mechanisms of oxidative stress and apoptosis in renal tubular epithelial cells are essential for the treatment of sepsis-associated AKI, little progress has been made in pharmacologic therapy. Renal tubular epithelial cells undergo necrosis and apoptosis *in vivo* and *in vitro* due to a variety of conditions, and this result was shown by cell morphology, apoptotic activation of cysteine asparaginase, and TUNEL assay for DNA damage (Thomas et al., 2022; Ren et al., 2020; Wang et al., 2020). And in several studies, baicalin is able to attenuate apoptosis in renal tubular epithelial cells through several pathways, such as mitochondria-dependent pathway, cell death receptor pathway (Wang Y. et al., 2018; Zhang et al., 2007). The caspase cascade triggers apoptosis in response to different injuries. Cacpase3 serves as the downstream effector in this cascade and initiates apoptosis directly upon activation by various upstream signals. Therefore, caspase3 is considered a key indicator (Yang et al., 2014; Yang et al., 2006). Bax and Bcl-2 are pro-apoptotic and anti-apoptotic proteins, respectively, in the mitochondrial apoptotic pathway. Because of their abnormal expression, the outer mitochondrial membrane becomes more permeable, allowing cytochrome c to pass through the membrane and into the cytoplasm (Spitz and Gavathiotis, 2022). In sepsis model mice, baicalin inhibits apoptosis by regulating Bax and Bcl-2 (Zhu et al., 2016). Cell FLICE like inhibitory protein (c-FLIP) is an apoptosis inhibitory protein that reduces lipopolysaccharide-mediated apoptosis in endothelial cells (Shen et al., 2017; Jang et al., 2017). Upon binding to FADD, the activation and recruitment of caspase-8 are inhibited by c-FLIP, thereby suppressing the cascade activation of downstream caspases and ultimately preventing apoptosis. A study demonstrated that via downregulating the expression of the negative regulator c-FLIP, c-Myc accelerates FasL/Fas-mediated apoptosis in renal tubular epithelial cells (Xu D. et al., 2019). In contrast, baicalin inhibited the apoptosis of renal tubular epithelial cells in SA-AKI mice, thereby considerably protecting the kidneys, and this protective effect may have been caused by enhanced c-FLIP protein expression (Le et al., 2021). Increased organ damage results from IRI, which is the stopping of blood flow to a specific organ and then resuming blood flow and oxygenation. Owing to its unique tissue structure and function, as well as its increased dependence on oxygen delivery, the kidney is especially susceptible to ischemia-reperfusion injury (Van Avondt et al., 2019). In a renal IRI model, baicalin lowered caspase-3 activity and decreased the Bax/Bcl-2 ratio, showing a favorable anti-apoptotic effect, thereby protecting the kidney and attenuating renal pathological changes (Lin et al., 2014a). However, the regulation of apoptotic signaling by baicalin is significantly more complex. For example, in a cellular model of arsenic-induced nephrotoxicity, baicalin elevated the Bcl-2/Bax ratio, thereby protecting the kidney and attenuating renal pathologic changes. The coexistence of necrosis and apoptosis in the pancreas, with necrosis being predominant, may explain why inducing pancreatic apoptosis results in a protective effect.

In AKI, cell death pathways cause necroinflammation (von Mässenhausen et al., 2018). Notably, unlike necrosis, apoptosis does not cause inflammation (Kønig et al., 2019). The term “pyroptosis” was first coined by Cookson and Brennan (2001) to refer to a cysteine-aspartate-specific protease 1 (caspase-1)-dependent cell death pattern. During pyroptosis, NLRP3 binds to the cysteine protease cysteine aspartate proteasome-1 to form inflammasome. Inflammasome can process pre-cysteine aspartyl protease-1 into mature cysteine aspartyl protease-1, leading to inflammatory death of a large number of somatic cells. Thus, “pyroptosis” is inflammatory death (Huang et al., 2021). Previous studies have identified IL-8 and IL-1β as significant markers of pyroptosis. Additionally, the cleavage and activation of the pore-forming effector protein gasdermin D (GSDMD) by activated cysteine asparaginase is a crucial step that triggers pyroptosis, leading to the compromised cells releasing IL-1β and IL-18 (Fu and Wu, 2023; Zhang X. et al., 2020). Apoptosis may result from pro-inflammatory cytokines activating immune cells, creating a detrimental cycle. Research has demonstrated that pyroptosis can be induced by contrast agents via the ROS/NLRP3/Caspase-1/GSDMD pathway, emphasizing baicalin’s capacity to block this mechanism. Intervention with baicalin attenuated the associated inflammation and oxidative levels (Li et al., 2022). Overall, Baicalin exerts an inhibitory influence on renal cell apoptosis by suppressing the activation of apoptotic signaling pathways. This protection shields renal cells from apoptosis triggered by diverse factors.

### 3.4 Baicalin prevents AKI by improving damage markers

In AKI, renal pathological changes include tubular injury, interstitial oedema, inflammatory cell infiltration, and more (Huang et al., 2020). Interestingly, baicalin attenuated renal pathological changes and improved renal histopathological scores in several studies, including models of sepsis-induced AKI (Le et al., 2021), or acute pancreatitis-induced AKI (Zhang et al., 2007; Zhang X. P. et al., 2008). For the most effective treatment to be given and additional kidney damage to be prevented, early identification of AKI is essential. In clinical practice, SCr, BUN and urine output are usually considered as the main indicators of renal function. SCr is a product of muscle metabolism, produced by muscle creatine metabolism, and SCr levels are readily available; however, Considering that creatinine concentrations are impacted by muscle mass, proteolytic metabolic rate, age, sex, and race, this is not a suitable marker. In addition, after AKI, SCr is a sluggish surrogate for decreased glomerular filtration rate, and it can take up to 72 h to return to a stable state. BUN is a waste product of protein metabolism, mainly converted from the conversion of amino acids to urea by the liver, both of which are excreted through the kidneys. When the glomerular filtration rate (GFR) decreases, the excretion of these two substances decreases, and the SCr and BUN levels in the blood increase accordingly (Macedo et al., 2011). Urine output and SCr are often the criteria for detecting AKI, but changes in his two are often inconsistent. Nonetheless, they are used as keys to diagnosing AKI (Kellum et al., 2013). A clinical study showed that when urea is reduced in patients with AKI, it reduces mortality (Chávez-Íñiguez et al., 2023). In studies on baicalin, it has been able to restore SCr and BUN to normal levels in multiple causes of AKI models (Wang et al., 2015; Chang et al., 2007). Notably, Clinical trials have demonstrated the considerable impact of baicalin. However, based on the pathogenesis and etiology of AKI, the original renal markers are limited. For example, volume expansion reduces SCr and delays recognition, whereas volume contraction induces an increase in serum concentrations, despite the absence of renal damage (Liu et al., 2011). Acute diseases (e.g., sepsis) and disease complications (e.g., severe malnutrition and sarcopenia) also reduce creatinine production. Neutrophil gelatinase-associated lipid carrier protein (NGAL), which is abundantly expressed and substantially upregulated in the kidney, is thought to be a novel biomarker with a high sensitivity for the detection of AKI (Shang and Wang, 2017). Although multiple renal injury markers can serve as important indicators for the detection of AKI, however, the markers due to different causes of AKI as well as different periods of AKI remain different. In critically ill patients and those with multiple comorbidities, identifying the underlying cause of AKI remains challenging. As an AKI biomarker, urinary NGAL, can help facilitate the differential diagnosis of chronic disease and intrinsic, pre-renal or post-renal etiologies at an early stage. As damage to the renal tubules is gradually repaired, the expression level of NGAL may decline (Romejko et al., 2023). Therefore, a drop in NGAL may indicate both a recovery of renal function and an improvement in renal tubular function. Research has demonstrated that baicalin can reduce NGAL level (Le et al., 2021). It is more sensitive than SCr, and When kidney cells are under stress or are damaged, this gene is expressed quickly. It is essential to identify AKI as soon as possible. Normal kidneys and other organs typically express the KIM-1 protein at low levels. However, its expression is notably upregulated in IRI rat kidneys and in rodent models of drug-induced AKI (Schrezenmeier et al., 2017). According to some authors, patients with AKI may experience histologic alterations prior to elevated Kim-1 levels (Zhang P. L. et al., 2008), implying that Kim1 could be one of the indicators for preferential detection of renal injury. In two studies on baicalin in the treatment of AKI, the authors detected changes in Kim1 reduction (Le et al., 2021; Shi et al., 2019). Although the above markers can be used to assess a certain degree of acute kidney injury, they all have limitations in diagnostic sensitivity and specificity, are susceptible to interference by multiple factors, and are still limited in early diagnosis and accurate assessment of the degree of injury. Hence, the pursuit of more precise and sensitive biomarkers remains a crucial objective in the investigation of AKI.

In conclusion, the anti-inflammatory, anti-oxidative stress and anti-apoptotic effects of baicalin in the treatment of AKI may be related to each other and jointly promote kidney protection. There is a close interaction between these three mechanisms, forming a complex network. For example, inflammatory responses can promote oxidative stress, and *vice versa*. After the release of inflammatory factors, ROS generation will be induced and the oxidative damage of cells will be aggravated. In addition, oxidative stress can also further activate inflammatory pathways, leading to apoptosis. Therefore, Baicalin can simultaneously reduce the occurrence of apoptosis by inhibiting inflammation and oxidative stress. Baicalin reduced ROS production by inhibiting inflammatory mediators, suggesting a positive feedback relationship between inflammatory response and oxidative stress (Li et al., 2022; Chen et al., 2024). Since oxidative stress can induce cell apoptosis, Baicalin reduces the risk of cell apoptosis by enhancing the activity of antioxidant enzymes, forming a virtuous cycle of antioxidant and anti-apoptosis (Li et al., 2022). The release of inflammatory mediators not only causes oxidative stress, but also directly promotes cell apoptosis. By inhibiting the expression of pro-inflammatory factors, Baicalin can effectively reduce the activation of apoptosis signal, thereby protecting the survival of kidney cells (Li et al., 2022; Zhu et al., 2016).

## 4 Protective effect of baicalin on DN and its renal fibrosis

Renal fibrosis can arise from CKD. The most typical route for the development of progressive renal disease is renal fibrosis (Martínez-Klimova et al., 2019; Nastase et al., 2018). The final stage of the development of many chronic kidney disorders is renal fibrosis, ultimately resulting in end-stage renal failure (Djudjaj and Boor, 2019). DN, a kidney disease brought on by consistently high blood glucose levels and a common side effect of diabetes mellitus, is a major contributor to the progress of end-stage renal disease (ESRD) (Association et al., 2003). In addition to kidney damage, people with diabetes often suffer from multiple kinds of complications such as neuropathy, retinopathyand cardiovascular disease. They are all at high risk of death (Rao Kondapally Seshasai et al., 2011). DN occurs in about 40% of diabetic patients and is characterized by proteinuria or reduced GFR (Viberti et al., 1982; Eknoyan et al., 2003; Levey et al., 2020). The exact mechanism of DN is unclear, and it has been show that uncontrolled hyperglycemia promotes oxidative stress, inflammation, reactivity, and fibrosis. The pathological definition of DN is the accumulation of extracellular matrix (ECM) in the glomerular mesangium, which thickens the basement membrane and eventually causes glomerulosclerosis (Samsu, 2021). At present, it is known that a variety of pathways play an important role in the occurrence and development of DN, such as TGF-β (Sharma and McGowan, 2000), Notch (Lin et al., 2010), Wnt (Rooney et al., 2011) pathway, and FGFR1, SIRT3, and dipeptidyl peptidase-4 (DPP-4) mediated signaling mechanisms also play an important role in the occurrence and development of DN (Liu Y. et al., 2022). Research findings have demonstrated that baicalin has the capacity to enhance renal function in DN patients and to mitigate the advancement of this condition by engaging multiple pathways, including anti-inflammatory and antioxidant mechanisms (Yang et al., 2019). In the latter case, the kidneys undergo an inadequate healing process; while they can recover from modest injuries, chronic or severe injury causes scarring (fibrosis) and renal function gradually declines (Hewitson et al., 2017). In turn, one of the key processes facilitating the shift from AKI to CKD is renal fibrosis (Romagnani et al., 2017). The main pathologic features of AKI and CKD are inflammation and fibrosis of the kidneys (Wu et al., 2022). Fibrosis is a warning that AKI is turning into CKD, so interrupting fibrosis is especially important.

**FIGURE 2:**
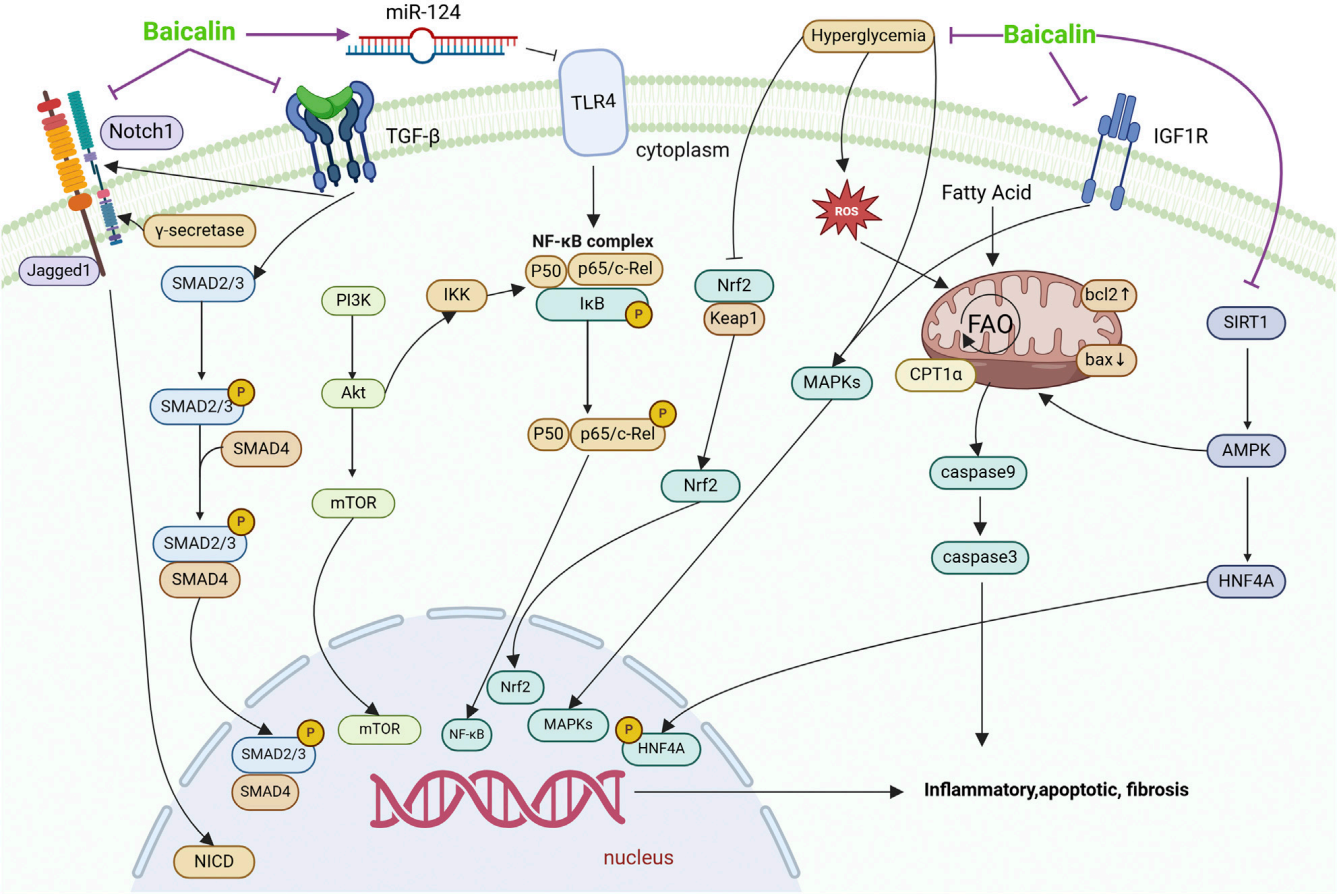
Mechanisms by which baicalin intervenes in renal fibrosis. This figure summarises the molecular pathways by which baicalin treats renal fibrosis. EMT, effector cell activation, local ischemia and hypoxia, and their effects on different signaling pathways cause renal fibrosis, leading to renal parenchymal damage, decreased glomerular filtration rate, and progression to chronic and ESRD. Baicalin affects fibrosis through TGF-β, Notch, IGF1R and TLR4 signalling pathways, which are involved in processes such as inhibition of inflammation, apoptosis, lipid metabolism and EMT.

As shown in Tables 2, 3, the studies of the baicalin for treating renal fibrosis are listed. From the perspective of mechanism, baicalin can lessen oxidative stress, mitochondrial damage, inflammation and apoptosis, and reverse matrix deposition and other cell protective mechanisms to alleviate renal fibrosis. These mechanisms are described in more detail below (Figure 2).

**TABLE 2:** The studies of baicalin intervention renal fibrosis *in vivo*.

Type	Animal/Cell	Experimental model	Dosages	Reno-protective effect	Mechanism	References
*In vivo*	Wistar rats	STZ-induced DN	Baicalin (75 mg/kg) and chrysin (10 mg/kg) combined	Antioxidant, anti-inflammatory	BUN↓, SCr↓, ROS↓, RAGE↓, CAT↑, GOx↑, SOD↑, GSH↑, GR↑, iNOS↓, PKC↓, p-IκB↓	Singh et al. (2017)
*In vivo*	Sprague-Dawley rats	UUO-induced renal fibrosis	Baicalin (20 and 40 mg/kg)	Anti-inflammatory	TGF-β1↓, Notch1↓, Jagged1↓	Tan et al. (2016)
*In vivo*	Sprague-Dawley rats	UUO-induced renal fibrosis	Baicalin (20 and 100 mg/kg)	Inhibits EMT	N- calmodulin↑, E− calmodulin↑, α-SMA↓, vimentin↓, TGF-β1↓, p-SMAD2↓, p-SMAD3↓	Zheng et al. (2017)
*In vivo*	Sprague-Dawley rats	STZ -induced DN	Baicalin (160 mg/kg/d)	Reduced SCr and BUN, anti-inflammatory	BUN↓, SCr↓, UPr↓, MDA↓, NF-κB↓, p-NF-κBp65↓, IL-1β↓, IL-6↓, IGF-R↓, mTOR↓, SIRT1↑, caspase-3↓, caspase-9↓, p-p38 MAPK↓	Zheng et al. (2020)
*In vivo*	Kunming mice	STZ -induced DN	Baicalin (40 mg/kg)	Reduced BUN and SCr and UACR, anti-inflammatory, anti-apoptosis, antioxidant, inhibits EMT	BUN↓, SCr↓, UACR↓, SOD↑, CAT↑, GPX↑, MDA↓, Nrf2↑, P65↓, TNF-α↓, NLRP3↓, CD3↓, CD68↓, IκB↑, Nrf2↑, caspase-9↓, TGF-β1↓, α-SMA↓, SMAD2/3↓, MMP13↑, β-catenin↓, E-Cadherin↑, Dnmt3a↓, Dnmt3b↓, Klotho↑	Zhang et al. (2020b)
*In vivo*	Sprague-Dawley rats	High-fat and STZ -induced DN	Baicalin (50, 100, 200 mg/kg)	Reduced BUN and SCr, anti-inflammatory	BUN↓, SCr↓, TC↓, TG↓,TNF-α↓, IL-1β↓, IL-6↓, LDH↓, SOD↑, MDA↓, p-PI3K/PI3K↓, p-Akt/Akt↓, p-mTOR/mTOR↓	Ou et al. (2021)
*In vivo*	db/db and db/m mice	Spontaneous DN model	Baicalin (40 mg/kg)	Antioxidant, anti-inflammatory	GSH-PX↑, SOD↑, CAT↑, MDA↓, TNF-α↓, IL-1β↓, IL-6↓, MCP-1↓, Nrf2↑, HO-1↑, NQO-1↑, U—Alb↓, ACR↓, AER↓, Bax↓, Bcl-2↑, Cleaved caspase-3↓, P38↓, Erk1/2↓, JNK↓	Ma et al. (2021)
*In vivo*	db/db and db/m mice	Spontaneous DN model	Baicalin (100 mg/kg/d)	Improves lipid metabolism	GLU↓, Alb↓, SIRT1↑, p-AMPKα↑, HNF4A↓	Zhang et al. (2022)
*In vivo*	C57BL/6 male mice	STZ -induced DN	Baicalin (15, 30, 45 mg/kg/d)	Reduced SCr and BUN, anti-fibrogenic, anti-inflammatory	BUN↓, SCr↓, ACR↓,COLIV↓, Fibronectin↓, miR-124↑	Zhang et al. (2020a)
*In vivo*	C57BL/6 male mice	UUO-induced renal fibrosis	Baicalin (10, 20, 40 mg/kg)	Reduced SCr and BUN, anti-inflammatory, anti-fibrogenic	BUN↓, SCr↓, α-SMA↓, COLA1↓, Fibronectin↓, TNF-α↓, IL-1β↓, IL-6↓, U—Alb↓	Wang et al. (2022)
*In vivo*	db/db and db/m mice	Spontaneous DN model	Baicalin (50 mg/kg/d)	Reduced BUN and SCr and UACR, improves lipid metabolism, anti-inflammatory	BUN↓, SCr↓, UACR↓, TCH↓, TG↓, HDL-C↑, LDL-C↓, ALB↓, α-SMA↓, Fibronectin↓, CPT1α↑, MCAD↑, Hmgcs2↑, PPARα↑	Hu et al. (2024)

**TABLE 3:** The studies of baicalin intervention renal fibrosis *in vitro*.

Type	Animal/Cell	Experimental model	Dosages	Reno-protective effect	Mechanism	References
*In vitro*	NRK-49F cells	TGF-β1-induced renal fibrosis	Baicalin (20, 40, 80 μM)	Anti-apoptosis, inhibits EMT	COLA1↓, COLA2↓, TGF-β1↓, SMAD3↓, SEAP↓,α-SMA↓	Hu et al. (2017)
*in vitro*	C57BL/6 male mice	HG-treated HK-2	Baicalin (100 μM)	Anti-inflammatory, anti-fibrogenic	COLIV↓, Fibronectin↓, p-IκBα↓, p-p65↓, TLR4↓, miR-124↑	Zhang et al. (2020a)
*in vitro*	HK-2 cells	0 mM glucose, and/or 100 ng/mL TNFα, and/or 100 μg/mL AGEs	Baicalin (5 μM, 10 μM)	Antioxidant, anti-inflammatory, anti-fibrogenic	FN↓, COL4A↓, TGFβ↓, ICAM1↓, VCAM1↓, IL1β↓, MCP1↓, pIκB↓, pJAK2↓, pSTAT3↓, cAMP↓, cGMP↓	Nam et al. (2020)
*in vitro*	HK-2 cells	HG-treated HK-2	Baicalin (50 μM)	Improves lipid metabolism, anti-inflammatory	TNF-α↓, IL-1β↓, IL-6↓, α-SMA↓, Fibronectin↓, CPT1α↑	Hu et al. (2024)

### 4.1 Antifibrotic effects of baicalin

Damage to tubular epithelial cells, glomerulosclerosis, inflammatory infiltrates, apoptosis, and interstitial fibrosis are typical pathologic characteristics of DN. The correlation between oxidative stress and DN has been well-documented in research. Research has demonstrated that mitigating oxidative stress can alleviate the manifestations linked to streptozotocin (STZ)-induced DN (Thallas-Bonke et al., 2008). Pathological and histological observations revealed that rats in the DN group had severe inflammatory lesions, glycogen deposition, and renal fibrosis, and treatment with baicalin had a significant ameliorating effect on these pathological changes. TGF-β has gained clinical recognition as a vital factor in the pathogenesis of ESRD (Meng et al., 2016) and has attracted widespread attention. Renal tubular epithelial cells can be differentiated into fibroblasts during the process of DN through various pathways (including autocrine and paracrine), accelerating tubulointerstitial fibrosis. As a major component of the cytoskeleton, α-SMA is important in the diagnosis and differential diagnosis of fibrosis. According to certain research, α-SMA is significantly expressed in the DN rats’ renal tissues, which can be used as an indicator of fibrosis detection (Zeng et al., 2019). Baicalin treatment significantly improved both renal fibrosis and renal function in this animal model by reducing TGF-β1 and α-SMA protein expression (Hu et al., 2017). The progression of DN is facilitated by TGF-β1 through its regulation of glomerular and tubulointerstitial fibrosis, a process that relies on Smad2 and Smad3 phosphorylation and activation. There is growing evidence that in the context of kidney injury, epithelial-mesenchymal transformation (EMT) occurs, in which renal tubular epithelial cells lose their phenotypic markers (including n-cadherin and e-cadherin) and transform into fibroblasts, myofibroblasts, or mesenchymal cells, acquiring mesenchymal phenotypic markers. Includes vimentin and α-SMA (Lan, 2003). In UUO-induced renal fibrosis model, baicalin significantly decreased the expression levels of α-SMA and vimentin, and increased the expression levels of N- and e-cadherin (Zheng et al., 2017). In STZ-induced DN, baicalin can inhibit EMT by partially regulating the methylation of klotho promoter, thus alleviating renal fibrosis (Zhang X. T. et al., 2020). In addition to canonical signaling pathways (Xianyuan et al., 2019). The ECM constitutes a fundamental element of the stromal and epithelial vascular matrix, encompassing collagen fibers (types I, III, and IV), non-collagenous glycoproteins (e.g., laminin, fibronectin), proteoglycans, elastin, and aminoglycans. TGF-β1 participates in both the synthesis and degradation of the ECM, thereby exerting a significant impact on the development of renal interstitial fibrosis (Yuan et al., 2022). Baicalin inhibits ECM accumulation by targeting the TGF-β1/Smad3 pathway (Zheng et al., 2017) and Notch signaling pathway (Tan et al., 2016) in rats with renal fibrosis. It also suppresses the p38 MAPK inflammatory signaling pathway and its downstream mediator NF-κB, thereby retarding the advancement of renal fibrosis (Zheng et al., 2020). Interestingly, though, one study found that via stimulating the TGF-β/Smad signaling pathway, high dosages of baicalin could instead cause kidney damage and fibrosis (Cai et al., 2017). The reason for this may be that it leads to accumulation of the drug, where the drug acts at other, non-specific targets, triggering unintended side effects or toxic reactions. Therefore, attention needs to be paid not only to individual differentiation but also to dosage issues during treatment.

### 4.2 Anti-apoptotic effects of baicalin

Research has demonstrated that DN patients who have prolonged hyperglycemia experience apoptosis and a deterioration in renal function. Additionally, renal tubular epithelial cells may undergo apoptosis in a hyperglycemic milieu (Ding et al., 2021). Tubular epithelial cells apoptosis is one of the main indicators of interstitial fibrosis and tubular atrophy (Johnson and DiPietro, 2013). Renal tubular epithelial cells from DN patients have been shown to exhibit apoptosis; however, fibrosis is delayed when renal tubular cell apoptosis is blocked (Huang F. et al., 2019). The processes of cell development, differentiation, death, environmental stress response, and inflammatory response are all impacted by activated p38 MAPK signaling (Coulthard et al., 2009). Research has demonstrated that renal inflammation and apoptosis are regularly regulated and promoted by p38 MAPK (Ren et al., 2020). In one study, Following treatment with STZ, there was a rise in caspase-3 and caspase-9 levels as well as hyperphosphorylation of p38 (Zheng et al., 2020). On the other hand, baicalin therapy decreased the production of apoptotic proteins in DN because these proteins strongly prevent apoptosis from starting in nephrotic tissues. This was accompanied by increased levels of insulin production with baicalin treatment, suggesting that baicalin may modulate the activation of the IGF-1/IGF-1R/p38 signalling pathway ameliorating STZ-induced renal fibrosis in DN rats (Zheng et al., 2020). Additionally, it has been discovered that JNK and p38, two members of the MAPK family, are upstream promoters of the mitochondrial apoptotic pathway. They can disrupt dimer formation and cellular localization to induce apoptosis through the mitochondrial pathway, or they can increase the Bax/Bcl-2 ratio (Chen et al., 2010). According to these research, the development of apoptosis and fibrosis in the kidneys is significantly influenced by p38 signaling.

### 4.3 Anti-inflammatory and antioxidant effects of baicalin

Numerous studies have demonstrated the critical roles that the development of diabetes is mostly influenced by inflammation and oxidative stress (Wang et al., 2019). Studies show that renal inflammation has a major role in the development and progression of fibrosis (Lv et al., 2018). Baicalin decreases renal fibrosis in DN patients by upregulating miR-124 and inhibiting the downstream TLR4/NF-κB pathway in STZ-induced mouse model and HG-induced HK-2 cell model (Zhang S. et al., 2020). In STZ-induced DN and UUO-induced fibrosis models, baicalin was found to significantly reduce inflammation levels, including TNF-α↓, IL-6, IL-1β↓and iNOS (Ou et al., 2021; Wang et al., 2022; Singh et al., 2017). Furthermore, in a model of spontaneous DN, baicalin attenuates aberrant lipid metabolism in the kidneys of db/db mice and may exert nephroprotective effects through the SIRT1/AMPK/hNF4A pathway (Zhang et al., 2022). Baicalin was found to decrease MAPK signaling in the same model, which enhanced Nrf2 activity and HO-1 production, lowering oxidative stress and slowing the progression of the disease (Ma et al., 2021). Other results together suggest that baicalin may protect against renal fibrosis by reducing inflammation-induced IκB phosphorylation, JAK2 phosphorylation, and subsequent NF-κB and STAT3 activation and oxidative stress in HK-2 (Nam et al., 2020).

### 4.4 Regulation of metabolic pathway of baicalin

In diabetes and other CKD, renal fibrosis is often associated with metabolic disturbances that may involve abnormalities in glucose, lipid and protein metabolism (Qu and Jiao, 2023). Baicalin may modulate the course of renal fibrosis by affecting these metabolic pathways. Currently, controlling the metabolism of lipids and glucose can help treat DN (Wang et al., 2023). However, these clinically standard therapies only slow the progression of the disease, not stop it. Therefore, another approach to the treatment of DN may include using drugs to activate the body’s cytoprotective pathways. Under normal conditions, diabetic kidney injury gradually develops in db/db mice on its own. A prolonged high glucose environment can lead to excessive proliferation and thickening of glomerular mesangial cells (GMC) and glomerular basement membrane (GBM) as well as damage to podocytes (Barutta et al., 2022; Tung et al., 2018; Tervaert et al., 2010). Proximal tubular epithelial cells are very important in renal function, They are in charge of various substances’ secretion and reabsorption, and an environment with high glucose levels may cause these cells to dysfunction, which can lead to renal fibrosis (Zhang et al., 2023; Liu L. et al., 2022). In STZ-induced mouse model and HG-induced HK-2 cell model, investigations were conducted into the effects of baicalin on renal fibrosis and its molecular mechanisms. Baicalin therapy boosted insulin production and ameliorated renal fibrosis via activating the IGF-1/IGF-1R/p38 signaling pathway (Zheng et al., 2020). Disorders of lipid metabolism, in addition to those of glucose metabolism, play a vital part in the onset and progression of DN (Xu et al., 2021). The transcription of lipid genes is regulated by the HNF4 family; among these, hNF4α controls several metabolic pathways in the kidney. Using molecular docking and network pharmacology, baicalin was discovered to bind to HNF4A and SIRT1 with efficiency (Zhang Q. et al., 2020), which indicates that baicalin may play a significant role in glucose and lipid metabolism. Further experiments showed that baicalin reduced triglyceride levels, and more importantly, baicalin was reported to attenuate lipid accumulation and MPC-5 in db/db mice by the SIRT1/AMPK/hNF4A pathway, suggesting that baicalin has a potentially attractive and potent therapeutic role in DN (Zhang et al., 2022). A vital enzyme in lipid metabolism, CPT1α, also known as carnitine palmitoyltransferase 1α, is a part of the fatty acid oxidation process. Long-chain fatty acids need to be converted to their corresponding carnitine derivatives before crossing the outer mitochondrial membrane, and this conversion is facilitated by the enzyme CPT1α (Lin et al., 2024). In the context of Diabetic kidney disease (DKD), according to mRNA sequencing, CPT1α activity is significantly suppressed, which causes a series of cellular disruptions that are essential for the development of renal fibrosis (Lin et al., 2024). According to a recent study, renal tubular cell damage was reversed and mitochondrial respiration was enhanced by up-regulating CPT1α, while down-regulating CPT1α increased renal tubular injury and interstitial fibrosis (Yuan et al., 2021). Intriguingly, Studies suggest that increasing CPT1α activity can prevent renal fibrosis from developing (Miguel et al., 2021). A study shows that baicalin targets CPT1α and enhances its expression to ameliorate impaired lipid peroxidation, thereby attenuating renal fibrosis in DKD (Hu et al., 2024). In addition, apoptosis of proximal tubular cells was also reported to be prevented by promoting CPT1α expression (Sun et al., 2018), therefore the relationship between baicalin and CPT1α, apoptosis deserves further investigation.

Similarly, multiple mechanisms play an important role in the occurrence and development of renal fibrosis. Anti-inflammation, anti-oxidative stress, anti-apoptosis and the regulation of material metabolism are related to each other in the treatment of renal fibrosis. Due to their correlation, baicalin can affect one pathway and then other pathways, such as inhibiting inflammation, inhibiting oxidative stress, and inhibiting apoptosis, which is extremely important in the treatment of renal fibrosis.

## 5 Constraints in utilizing baicalin clinically and strategies for enhancing its efficacy

**FIGURE 3:**
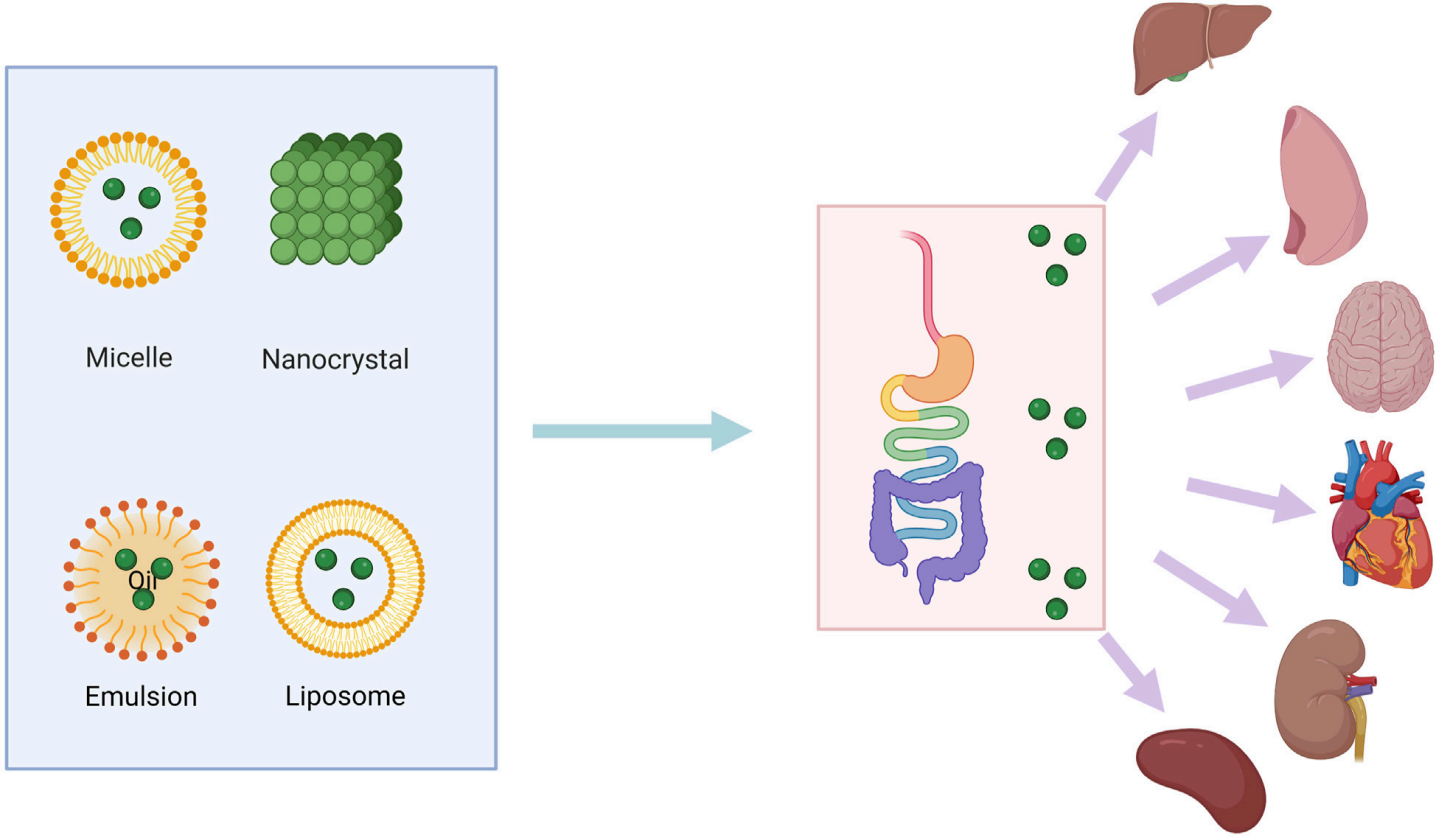
Various methods for increasing bioavailability of baicalin. The green dots represent the natural drug baicalin, which enters the body in liposomes or other carriers. It is digested and absorbed through the intestines into various parts of the body, including the liver, kidneys, lungs, and spleen.

Baicalin’s pharmacological effects have been verified through a variety of ways. However, baicalin has poor water and fat solubility, while generally speaking, well-absorbed chemicals have strong water and fat solubility. One study showed that baicalin belongs to Class IV of the biotherapeutic classification scheme due to its very low hydrophilicity (solubility in water 0.052 mg/mL) and lipophilicity (Papp = 0.037 × 10^-6^ cm/s), resulting in very low bioavailability of baicalin due to poor solubility (Wu et al., 2014). This could be due to flavones and glucuronide could compose intramolecular hydrogen bonds that lead to the low solubility, which results in low oral bioavailability (2.2% ± 0.2% in rats) (Wu et al., 2018). Similarly, baicalin has poor lipophilicity, so it is not easily absorbed as a mother. In addition, baicalin also has unique characteristics of gastrointestinal absorption and biotransformation, including hydrolysis of intestinal bacteria, undergoing enterohepatic circulation, and gluconaldehyde acidification (Chen et al., 2014). Its bioavailability is low due to the aforementioned characteristics, and a higher dose is frequently needed to produce a favorable therapeutic effect. At the same time, its toxicity should also be taken into consideration. Its clinical usefulness is occasionally limited by its low bioavailability. As a result, increasing baicalin’s bioavailability is crucial. Newly created baicalin preparations with improved absorption and increased bioavailability are the result of advancements in preparation processes. In Figure 3, we have enumerated the innovative tactics and provided an overview of the study effort toward enhancing baicalin’s bioavailability. Drugs can be made into nano-emulsions, nano-suspensions, nano-solid lipid nanoparticles and nano-lipid carriers by nanotechnology. When the drug particle size reaches nanoscale, due to the effect of quantum size and surface effect, nanoparticles show new physical and biological properties, thereby improving the biological activity and bioavailability of drugs. It can overcome many defects of traditional medicine. For medications like baicalin that are poorly soluble, liposomes are an effective drug delivery mechanism because they increase the solubility and stability of the molecule. Baicalin-liposomes enhanced the tissue distribution and oral bioavailability of baicalin in comparison to baicalin solutions (Huang T. et al., 2019). The optimal prescription was as follows: phospholipid drug ratio 3.81:1, phospholipid cholesterol ratio 5.70:1, hydration volume 1.02 mL, ultrasonic power 60W, ultrasonic time 10 min, ultrasonic temperature 20°C ± 5°C successfully prepared liposomes. A study on the development of BA-loaded liposomes (BAI-LP) showed a significant increase in drug concentrations of BAI-LP in the liver, kidney, and lungs (Li et al., 2018), indicating that BAI-LP might be a viable oral medication administration method to increase baicalin’s bioavailability. In addition to improve utilization, liposome-encapsulated baicalin also improves drug targeting. A study successfully used lipids/cholesterol (L), long circulating invisible liposomes (L-PEG) and folate receptor (FR) -targeted liposomes (L-FA) as drug carriers for baicalin. By modifying folic acid on the surface of the liposomes, it can improve its affinity to specific cells, thereby enhancing its efficacy. The finding demonstrated increased cytotoxicity and intracellular absorption of baicalin liposomes with folate receptor (FR) targeting in comparison to non-targeted liposomes, indicating that baicalin loaded FR-targeted liposomes (L-FA-BAI) could improve the anti-tumor impact (Chen et al., 2016). Baicalin’s oral bioavailability can be greatly increased by baicalin-loaded nanoemulsions because of their sustained-release properties (Zhao et al., 2013). However, liposomes may degrade while being used and stored, which could reduce the drug’s efficacy. One significant problem is figuring out how to regulate the rate and timing of drug release. To attain the intended drug release characteristics, researchers must adjust the liposomes’ structure and content. Nanoemulsion is a transparent, thermodynamically stable mixture of water, oil, co-surfactant, and surfactant. Nanoemulsion is a dispersion with droplets smaller than 100 nm that is stabilized by an interfacial layer (Wu et al., 2009). A novel nanoemulsion improves systemic exposure to baicalin. Study using celiac flow blockage and *in situ* single-pass enteral perfusion indicate that intestine absorption and lymphatic transport play a role in systemic exposure to baicalin (Wu et al., 2018). Baicalin’s lymphatic system concentration can be increased using nanoemulsions. A baicalin nanoemulsion demonstrated enhanced bioavailability in the transfer of baicalin to the lymphatic system, according to an *in vivo* investigation (Xu Q. et al., 2019). The mixed micelle system is a micron-scale structure composed of two or more different surfactants self-assembled in the water phase, and its characteristics give it special properties. For example, the outside of the micelle is covered by water-soluble polar groups, while the hydrophobic alkyl group portion is hidden inside. This structure makes the mixed micelles exhibit good stability and biocompatibility in the interaction with the water phase (Carey and Small, 1970). By coating drug molecules, the mixed micelle can improve the bioavailability and therapeutic effect of drugs (Ezhilrani et al., 2021). After oral administration, the BAI-loaded mixed micellar system containing sodium taurocholate and Pluronics P123 copolymer as carrier materials exhibited a sustained release effect. Oral bioavailability was also enhanced, and compared to free baicalin solution, Its absorption through the intestinal tract was noticeably greater (Zhang et al., 2016). Nonetheless, research on the biocompatibility and intracellular absorption of mixed micelles is still crucial for drug delivery applications. Current biodegradable micelles’ therapeutic efficacy is impacted by issues such sluggish intracellular drug release and limited tumor cell uptake (Zhong et al., 2013). The self-emulsifying drug delivery system (SMDDS) can make lipophilic drugs spontaneously form a stable microparticle system in water, improve their dissolution and dispersion ability, and promote drugs to pass through intestinal epithelial cell mucosa to improve bioavailability. SMDDS dissolve hydrophobic drugs in the oil phase and spontaneously form emulsions upon contact with the water phase. To enhance baicalin oral absorption, phospholipid complex (PC) and SMDDS can work in concert. Single-pass intestinal perfusion models and Caco-2 cell uptake studies shown that this formulation might greatly increase baicalin transit and relative bioavailability (Wu et al., 2014). It is noteworthy that the properties of compounds can be improved by structural modification (Yao et al., 2017). According to two recent research, the substitution and esterification of baicalin by halogenated hydrocarbons can lead to the synthesis of baicalin-2-ethoxyethyl ester, a new compound that significantly improves the low bioavailability and low solubility of baicalin, and has been discovered to significantly ameliorate AKI and renal fibrosis in mice *in vivo* experiments (Chen et al., 2024; Li et al., 2024). Although structural modification can increase baicalin’s bioavailability, there are drawbacks to this as well. A large modification of a structure may prevent its binding to the target molecule, which may affect its biological activity and efficacy (Wen et al., 2023). There are two challenges in this area that need further exploration. First, while a large portion of research has focused on improving nanotechnology-based bioavailability, few studies have provided scientific explanations or explanations for how baicalin’s bioavailability is genuinely enhanced by nanoformulations. Secondly, although some have reported significant enhancements or modifications to ADME characteristics, comparatively little study has been conducted on further uses of nanomaterials.

## 6 Clinical prospects

Clinical trials are very important for drugs to provide key information on efficacy, safety and dosage to provide a scientific foundation for clinical application of drugs. Clinical practice makes extensive use of baicalin, which is isolated from Scutellaria baicalensis. In one study, Baicalin was administered orally to 374 patients suffering from rheumatoid arthritis and coronary artery disease at a dosage of 500 mg/d. The patients’ blood lipid levels and inflammation were found to be reduced by baicalin (Hang et al., 2018). In addition, baicalin reduced the survival of peripheral blood leukocytes (PBLs) in individuals suffering from acute lymphoblastic leukemia (ALL), increased IFNγ production in PBLs and decreased the production of TNFα and IL-10 in bone marrow cells (BMCs) in ALL patients. Importantly, baicalin did not induce PBLs in healthy controls to undergo apoptosis (Orzechowska et al., 2014). In addition, baicalin can be combined with other herbal extracts to form a drug to reduce the severity of postoperative pain after impacted mandibular third molar surgery (Isola et al., 2019). In AKI, a study of SCr and BUN levels in 50 pediatric patients with or without baicalin-assisted therapy found that baicalin-assisted therapy significantly reduced SCr and BUN levels in children with sepsis (Zhu et al., 2016). However, it is significant to note that further research is needed to completely evaluate the safety, effectiveness, and ideal dosage of baicalin in these indications. This research should include longer-term studies as well as larger clinical trials.

Drugs similar to baicalin, such as baicalein, have been shown to be safe in a single oral dose of 100–2,800 mg of baicalein in healthy people (Li et al., 2014). In dose range of 200–800 mg, multiple-dose oral baicalein administration was safe and well tolerated, dose proportionality was inconclusive, and no serious accumulation of baicalein was observed (Pang et al., 2016). Breviscapine is a flavonoid that also has a favorable protective impact on the kidney, can lower blood pressure, and enhance renal function and urine microalbuminuria (Wei and Tan, 2005). In addition, Breviscapine has many clinical indications, mainly used in the treatment of coronary heart disease, angina pectoris, hypertension, hyperviscoemia and cerebral ischemia. Therefore, we speculate that baicalin, which is also a flavonoid, can enter the clinic and has certain safety.

Currently, multi-drug combination therapy is now common in many diseases and can lead to drug interactions (Cheng et al., 2019). The interaction between baicalin and other drugs is also noteworthy. In kidney disease, commonly used drugs are antibiotics, blood pressure drugs such as nifedipine, immunosuppressants such as cyclosporin A and so on. The study found that gut flora plays a key role in drug absorption and utilization (Feng et al., 2019). The effectiveness of baicalin can be impacted by gut bacteria and β-glucosidase structurally converting it to baicalein (Xing et al., 2005b). Therefore, when baicalin is combined with antibiotics, the effect of antibiotics on the pharmacokinetic properties of baicalin by inhibiting intestinal bacteria should be considered. As natural carriers of many endogenous and exogenous drugs, plasma proteins are responsible for determining the pharmacokinetic properties of many drugs. The plasma protein binding rate of baicalin was found to be between 86% and 92%, suggesting that baicalin is a competitive substitute for some drugs and plasma protein (Tang et al., 2006). In a randomised, three-period crossover study, researcher evaluated the pharmacokinetics of nifedipine and baicalin (0.225 or 0.45 g/kg) after intravenous administration in rats. The results showed that the pharmacokinetics of nifedipine could be significantly changed: at low doses, the total concentration maximum (C_max_) of nifedipine was decreased by 40%, the area under the concentration–time curve (AUC_ ∞ _) was decreased by 41%, the volume of distribution (Vd) was increased by 85%, and the clearance rate (CL) was increased by 97%. At high doses, Cmax was reduced by 65%, AUC_ ∞_ by 63%, Vd by 224%, and CL by 242%. At the same time, C_max_ of free nifedipine increased by 25% at low doses and 44% at high doses, suggesting that baicalin may regulate the pharmacokinetics of nifedipine by affecting plasma protein binding and inhibiting cytochrome P450 3A (CYP3A) activity (Cheng et al., 2014). CYP3A subfamily and P-glycoprotein (P-gp) exist in human intestinal epithelial cells at high levels as metabolic enzymes and efflux transporters, respectively. At the same time, the substrate of P-gp overlaps that of CYP3A4 (Morisaki et al., 2013). As a substrate of P-gp and CYP3A4, the C_max_ and AUC_0-540_ of cyclosporine increased significantly by 408.1% and 685.3%, respectively, after it was combined with baicalin. The pharmacokinetic parameters of rats treated with baicalein (112 μmol/kg) were also increased by 87.5% and 150.2%, respectively. However, there was no difference in the clearance stage of cyclosporin regardless of baicalin or baicalein treatment (Lai et al., 2004). These results indicate that it is necessary to consider the effects of drugs on each other, although both drugs have positive effects on the disease.

## 7 Conclusion and future perspective

Baicalin, as a potential herbal active ingredient for the tre atment of AKI and renal fibrosis, possesses wide range of pharmacological activities and multiple mechanisms of action. Baicalin exhibits a protective effect on the kidney in AKI by lowering oxidative stress, decreasing the inflammatory response, and preventing the release of inflammatory cytokines. It also reduces necrosis and apoptosis of renal tubular epithelial cells and promotes tubular repair. For DN and its renal fibrosis, baicalin can act through several pathways. It can lessen the synthesis and accumulation of ECM and suppress the fibrotic and inflammatory reactions of renal tubular epithelial cells brought on by hyperglycemia. In addition, baicalin can intervene in diabetes-related signaling pathways, such as TGF-β1, thereby reducing the process of renal fibrosis. In clinical trials, baicalin was able to show better results. However, there are some limitations in the current research on Baicalin, including insufficient pharmacokinetic studies and limited clinical trial data.
